# General Medicine and Therapeutics

**Published:** 1893-09

**Authors:** 


### GENERAL MEDICINE AND THERAPEUTICS.

EDITED BY DR. LUTHER B. GRANDY.

### LIMITED IMPORTANCE OF ALBUMINURIA IN THE DIAGNOSIS OF BRIGHT'S DISEASE.

In a recent address delivered before the Academy of Medicine of Paris, Dieulafoy tried to prove that too much importance is given to albuminuria in establishing a diagnosis of Bright's disease and gauging its gravity, basing his opinion upon observations in sixty cases taken from his hospital wards, and duly classified.

In the first category were included six cases in which death followed all the major signs of uremia, after having presented most of the minor signs; no albumin, however, had been found at any time, although the post-mortem examination demonstrated the existence of characteristic lesions of Bright's disease.

In a second group he placed certain cases in which albumin was found at certain periods only, outside of which periods it was impossible to diagnose chronic nephritis by searching only for the pathognomonic symptom, albuminuria. Dieulafoy therefore concluded that this symptom had but little value in the diagnosis of Bright's disease. It should only serve to confirm the presence of the disease as corroborative evidence; for, being far from trustworthy, it might otherwise lead to a diagnosis of Bright's disease in subjects having had albumin a long time without showing signs of ill health. To avoid having recourse to a symptom proven to be so untrustworthy, this illustrious observer proposes that one should make use of those "minor signs," for the most part unperceived generally, which he was the first to describe under that name in his works on the subject. These minor signs, according to him, serve to indicate the initiatory stage of Bright's disease, the malady itself being characterized by the major signs, such as dyspnœa, cephalalgia, convulsions. He describes, in turn, the symptoms furnished by the sense of hearing—the buzzing and whistling varieties of tinnitus so frequent in cases of Bright's disease; the strange sign that he characterized as the "doigt mort" (dead finger), characterized by a sensation analogous to that produced by putting one's fingers in the snow, itching, etc., etc. He also speaks of cryæsthesia, that sensation of feeling cold peculiar to victims of Bright's disease; slight epistaxis, in the morning, and also the painful cramps in the legs which only cease on the patient's getting out of bed, are also symptoms to be noted.

Dieulafoy has always found these minor signs of use in the diagnosis of chronic nephritis, and had them controlled by measuring the degree of toxicity of the urine of patients; he always found a smaller degree of toxicity than is normal, this serving to prove that the substances to be eliminated by the kidneys remained in the system to gradually poison it.

As to treatment, Dieulafoy considers that milk diet is the only effective means at our disposal. He does not attach much importance to the variations in the quality of albumin excreted while under the influence of this treatment, as the improvement—the cure, even, of certain patients—is shown by the disappearance of the minor signs, sometimes even by that of the major symptoms. This he has witnessed in two groups of patients, where the two pathological conditions were associated—Bright's disease and chlorosis on the one side, and Bright's disease and syphilis on the other.—*Universal Medical Journal.*

---

### A LAY VIEW.

In these days when the *Sun's* reporters are writing learnedly of the comma bacillus, those of the *Whirled* airing their ignorance of the nature of Asiatic cholera, the *Herald* and the *Recorder* making a lively race for third place in asininity regarding our annual "summer complaint" scare, and even the so-called bacteriologists of the city's medical staff talking as though the study of cholera had been reduced to an exact science, it appears to me wise to call my readers' attention to the limitations of knowledge of the most fundamental sort that characterizes the medical profession on this subject. This I do with no intention to shake the public's faith in their medical advisers, but to show that this whole matter of cholera epidemics and of the deadly inroads of the comma bacillus is largely one of collective fright and the consequent stampede, and one in which, after all is said and done, correct living and decent habits are the all-powerful factors which make for health.

In the first place let it be understood that the comma bacillus—so generally alluded to as the *cause* of cholera—has frequently been found in the mouths or in the intestines of perfectly healthy persons and of other animals. So, too, they have been found in the intestines of persons who have died of other diseases in no way related to cholera. Professor Waters has well-nigh satisfactorily indicated that this much-feared microbe "is a harmless and even necessary inhabitant of the animal economy." And, as pointed out by Bulman, there seems some support for the startling suggestion that the microbe, which has been a source of alarm to the civilized world, may, after all be a necessary aid to digestion! Chapman, an author of no mean pretensions, calls attention to the fact that in India, cholera's home, it is regarded by the medical profession as non-contagious, and cholera patients are treated in the wards along with other patients quite as successfully as under the European procedure, as I can affirm from my own observation. Again, the considerable immunity of cholera nurses, and the fact that students, who in cholera times have little else than cholera patients for dissection, are not thereby affected with cholera, point still more strongly to the conclusion that the bacillus stampede, as it may well be called, has been founded on the most unscientific data. In saying which, it will be understood, I have but stated the case for the bacillus; the other side has been printed and preached *ad nauseam*. From this presentment it will be still more clear than heretofore that with the enemies of the bacillus valiantly opposing its entrance to our port and the adherents of the doctrine of the nervous nature of the disease showing mankind how essential are correct habits and a cheerful mind, the cholera has even less chance than it had last year of setting its foot on Manhattan Island.—"The Saunterer," *Town Topics*, New York.

